# Signal-to-Noise Ratio Enhancement Based on Empirical Mode Decomposition in Phase-Sensitive Optical Time Domain Reflectometry Systems

**DOI:** 10.3390/s17081870

**Published:** 2017-08-14

**Authors:** Zengguang Qin, Hui Chen, Jun Chang

**Affiliations:** School of Information Science and Engineering and Shandong Provincial Key Laboratory of Laser Technology and Application, Shandong University, Jinan 250100, China; hui931001@163.com (H.C.); changjun@sdu.edu.cn (J.C.)

**Keywords:** vibration location extraction, phase-sensitive optical time domain reflectometry, empirical mode decomposition, Pearson correlation coefficient, signal-to-noise ratio

## Abstract

We propose a novel denoising method based on empirical mode decomposition (EMD) to improve the signal-to-noise ratio (SNR) for vibration sensing in phase-sensitive optical time domain reflectometry (φ-OTDR) systems. Raw Rayleigh backscattering traces are decomposed into a series of intrinsic mode functions (IMFs) and a residual component using an EMD algorithm. High frequency noise is eliminated by removing several IMFs at the position without vibration selected by the Pearson correlation coefficient (PCC). When the pulse width is 50 ns, the SNR of location information for the vibration events of 100 Hz and 1.2 kHz is increased to as high as 42.52 dB and 39.58 dB, respectively, with a 2 km sensing fiber, which demonstrates the excellent performance of this new method.

## 1. Introduction

Distributed fiber optical sensors are an effective technology for measurement of physical quantities such as strain, temperature and vibration. They have been widely applied in many fields, including health monitoring of engineering structures, security and mechanical processes. Configurations based on interferometers, including Sagnac interferometers, Mach-Zehnder interferometers and Michelson interferometers [1,2,3,4] have been introduced to measure external intrusions along the fiber, which are limited by low spatial resolution and intricate data processing. Distributed fiber sensors based on backscattering light along the fiber include polarization-OTDR, phase-sensitive OTDR (φ-OTDR) and Brillouin OTDR [5,6,7,8,9] devices are proposed to locate the position of disturbances with a satisfactory spatial resolution. In recent years, all these systems have seen significant developments for enhancing their performance, especially φ-OTDR. Ultra-long sensing distance of 131.5 km was realized in φ-OTDR with high spatial resolution by combining heterodyne detection and Raman amplification [10]. The use of different laser frequencies in φ-OTDR was proposed to measure multiple parameters simultaneously [11]. The waveform and amplitude of vibrations can be recovered by distinguishing the differential phase in the sensing fiber, which is a prominent advancement in φ-OTDR systems [12]. Therefore, φ-OTDR is regarded as a promising technology worthy of further study, with the advantage of a fully distributed manner, high detection sensitivity and fast response.

In φ-OTDR systems, the laser must have a narrow linewidth and low-frequency drift to ensure the coherence of light. Repetitive optical pulses are injected into the sensing fiber and the Rayleigh backscattering curves returning from the fiber are influenced in the form of a speckle-like profile [13] due to the coherent effect of multiple scattering points within the pulse duration. Note that the amplitude of the Rayleigh backscattering curves is theoretically constant at each position along the fiber as long as the scattering points do not change. Disturbances such as strains or vibrations can affect the refractive index of the sensing fiber, thus the amplitude of Rayleigh backscattering curves corresponding to the disturbance position will be changed. The location of external disturbance can be determined by distinguishing the variations among the curves. However, the Rayleigh backscattering light is susceptible to the surrounding environment in practice because of its high sensitivity. On the other hand, random noises such as phase noise of the laser and electrical noise can deteriorate the signal and make vibration detection more difficult. Many works on locating disturbances and enhancing the SNR of φ-OTDR systems have been carried out. The moving average and moving differential methods are demonstrated to reduce the amplitude fluctuation in Rayleigh backscattering traces [14]. Detection performance of vibration measurement is improved and the signal is extracted from the background noise by using wavelet denoising methods [15]. Application of a two-dimensional edge detection method in φ-OTDR systems is proposed, and the SNR is increased to 8.4 dB with a pulse width of 50 ns [16]. A high-performance way based on power spectrum analysis is introduced to eliminate undesirable effects resulting from frequency drifts of the lasers and this improves the SNR of φ-OTDR systems [17]. The features of Rayleigh backscattering traces themselves in the φ-OTDR system may not be considered in all these signal processing methods mentioned above. All the raw data at different positions with or without the vibration event along the sensing fiber is handled in the same way. Therefore, the characteristics of the raw signal with or without the vibration are totally ignored, which may lead to poor SNR in the φ-OTDR system.

In this paper, a novel denoising method in which the inherent characteristics of the Rayleigh backscattering signals are considered during the denoising process is introduced in φ-OTDR systems for the first time. The cluster of Rayleigh backscattering traces is decomposed into a series of IMF components and a residual component. A criterion based on the Pearson correlation coefficient is proposed to distinguish the position with or without the vibration event. Performance comparisons with other denoising methods are performed. Vibration events of 100 Hz and 1.2 kHz are experimentally demonstrated and the SNR of location information is improved to 42.52 dB and 39.58 dB, respectively, with a pulse width of 50 ns and an optical fiber length of 2 km.

## 2. EMD Denoising Method

The EMD algorithm was introduced by Huang et al. for non-stationary and non-linear data analysis [18]. This method is used to decompose a noisy signal into a series of basis functions which are designated as intrinsic mode functions (IMFs) and a residual component. For a given noisy signal *x(t)*, it is shown as follows:

(1) $$x(t)=\overset{N}{\underset{j=1}{\Sigma }}IMF_{j}(t)+r_{N}(t)$$

**Figure 1:**
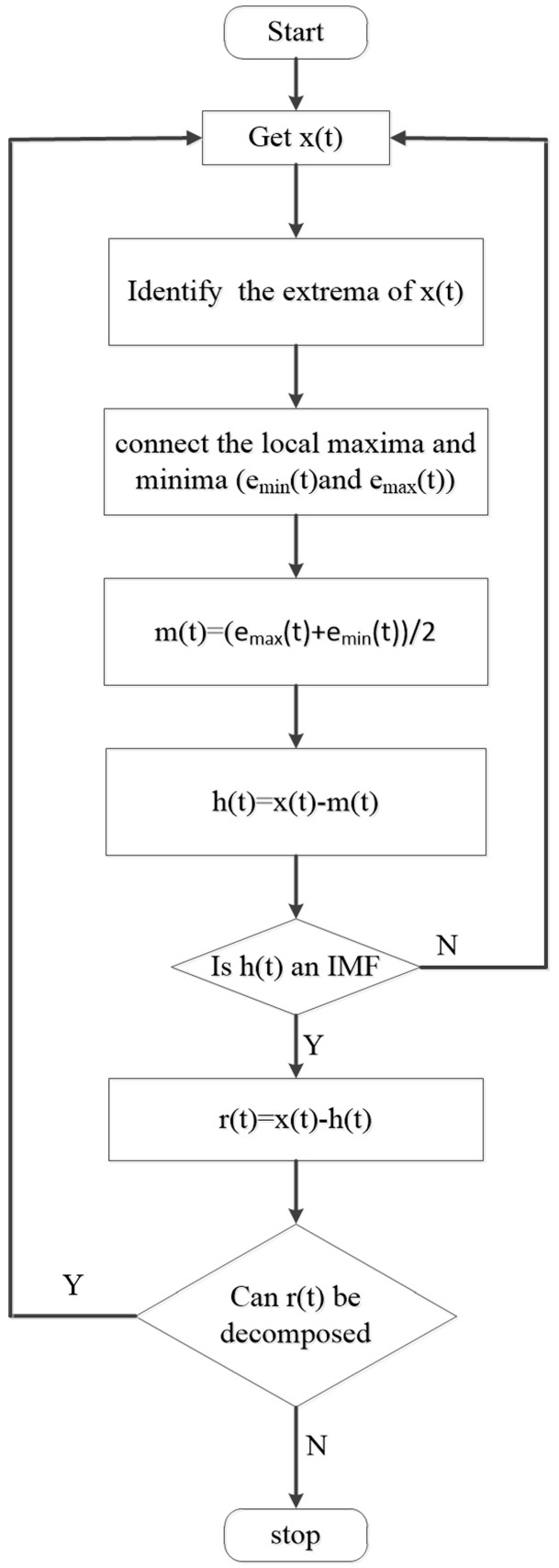
Block diagram representing the five procedures to achieve EMD.

In the equation, where *N* is the number of IMFs, *r_N_*(*t*) is the residual component. Actually, most of the data cannot decompose into IMFs directly which must satisfy two conditions [18]. A process called sifting is proposed to solve this problem shown in Figure 1 and described as follows:

(1) Identify the extrema of *x*(*t*).
(2) Connect the local maxima and minima by a cubic spline as the upper and lower envelopes respectively, which should involve all the data between them.
(3) Calculate the mean of two envelopes designated as *m*(*t*).
(4) Compute the difference between *x*(*t*) and *m*(*t*) and get the first component *h*(*t*), h(t)=x(t)−m(t).
(5) If *h*(*t*) is an IMF, compute the difference between *x*(*t*) and *h*(*t*) and get the first residual component *r*(*t*). *R*(*t*) is treated as the *x*(*t*) and repeat step 1 to 5 to acquire the surplus IMFs. Otherwise, *h*(*t*) is treated as the *x*(*t*) and repeat step 1 to 5 until it is an IMF.

Note that there are concrete conditions to judge Step 5 in order to achieve EMD in the computer program [19]. The number of IMFs is decided by the data itself during the sifting process. The IMFs obtained by the sifting process have the characteristic that their frequency decreases gradually from the first (IMF1) to the last, so the noise characterized by high frequency is mainly concentrated on the IMF1 and decreases toward to the last IMFs. Therefore, this method can be used for noise reduction by filtering or thresholding some IMFs [20,21,22,23]. 

The Pearson correlation coefficient (PCC) [24] is a parameter to quantify the correlation of two variables. Computing the PCC between *x*(*t*) and IMFs is beneficial to obtain the particular mode which IMF is dominated by signal rather than noise. The PCC is defined as:

(2) $$PCC=\frac{\sum_{i=1}^{n}\left(x_{i}-\bar{x}\right)\left(y_{i}-\bar{y}\right)}{\sqrt{\left[\sum_{i=1}^{n}{\left(x_{i}-\bar{x}\right)}^{2}\right]\left[\sum_{i=1}^{n}{\left(y_{i}-\bar{y}\right)}^{2}\right]}}$$

where *x_i_* and *y_i_* are the two variables, whereas $\bar{x}$ and $\bar{y}$ are the arithmetic mean of the two variables. *n* is the length of the variables.

**Figure 2:**
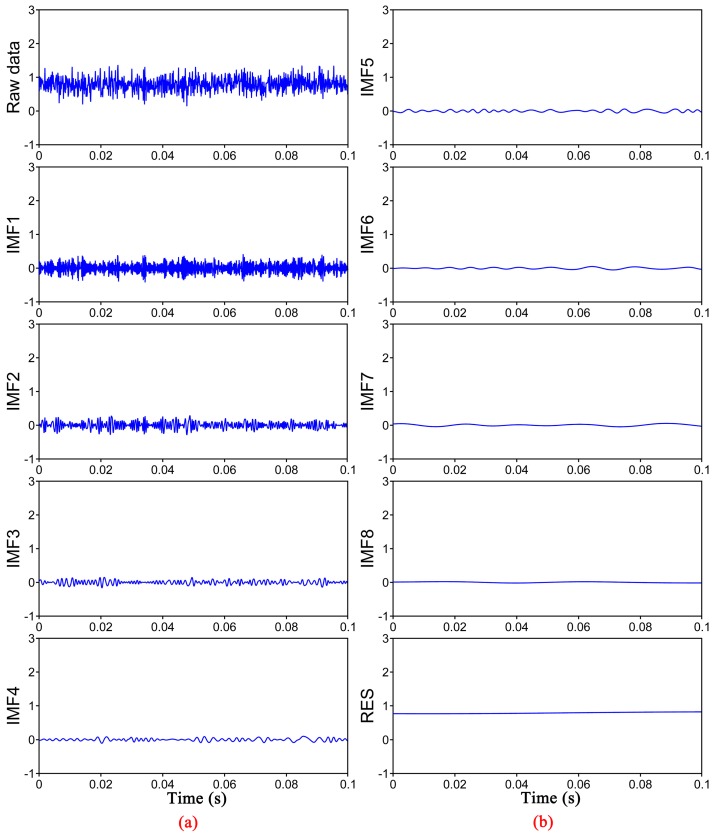
The resulting EMD components from the raw data (**a**) The raw data and the components IMF1-IMF4; (**b**) The components IMF5-IMF8 and RES.

**Figure 3:**
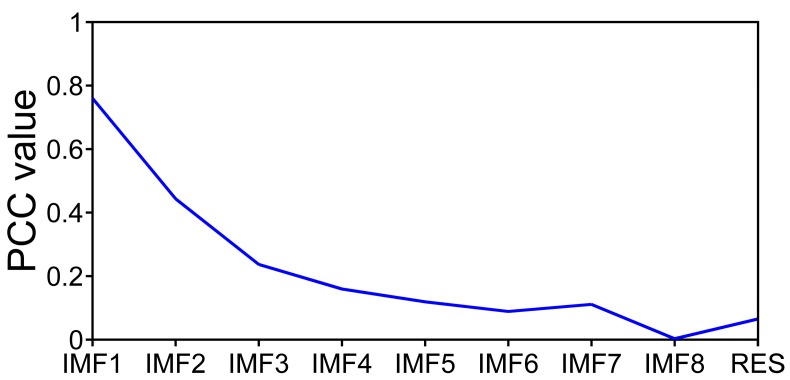
PCC between raw data and each IMFs.

In φ-OTDR system, because of the interference effect of Rayleigh backscattering light, there are direct-current components with different amplitude along the sensing fiber. Meanwhile, the raw data only consists of a direct-current (DC) component and random noise at the fiber section without a vibration event. The Rayleigh backscattering signal at the vibration point is modulated by the external disturbance and superposed by random noise just like the position without the vibration. The EMD combined with PCC (EMD-PCC) denoising method is proposed in view of the feature of Rayleigh backscattering traces acquired from φ-OTDR system. We set up two simulation experiments to confirm the feasibility of the denoising method. A simulation experiment is based on the raw data consisted of a DC component with amplitude of 0.8 V and Gaussian white noise with the amplitude of 0.2 V. Figure 2 shows the components obtained by EMD from the raw data. It can prove that from IMF1 to RES, the frequency information decreases gradually. Meanwhile, we cannot recognize the DC component from the raw data, while the appearance is decided by the noise. In order to denoise the DC signal, we calculate the PCC between raw data and each IMFs, shown in Figure 3. It is observed that the PCC between raw data and IMF1 is the maximum. In this situation, we deduce that the DC component is recovered by only retaining the residual component which is fluctuated with the center of 0.8 V as shown in Figure 2.

**Figure 4:**
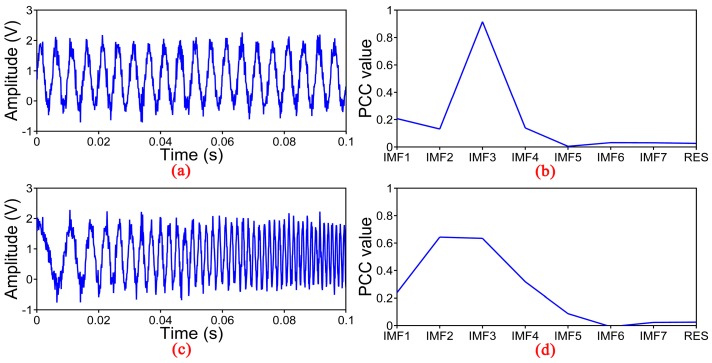
Another simulation experiment with different vibration events (**a**) The first vibration events consisted of a DC component, Gaussian white noise and sinusoidal signal; (**b**) PCC between the first vibration events and each IMFs; (**c**) The second vibration events consisted of a DC component, Gaussian white noise and chirp signal; (**d**) PCC between the second vibration events and each IMFs.

Figure 4 shows the simulation results with different vibration events including sinusoidal and chirp signals corresponding to the position with the vibration in the φ-OTDR system. Sinusoidal signal with the frequency of 200 Hz and the amplitude of 1 V mixed with the interference mentioned above is shown in Figure 4a. We can see that the appearance is determined by the sinusoidal signal. Figure 4b shows the PCC between the noisy signal and each IMFs. The maximum of the PCC appears at the position of IMF3 which is different from the first simulation experiment. The chirp signal with the frequency range from 50 Hz to 800 Hz is mixed with the DC component and noise, shown in Figure 4c. In Figure 4d, there are two large PCC values appear at the position of IMF2 and IMF3. Different IMFs contain information within different frequency ranges, so this multi-frequency signal information can be found in multiple IMFs. Anyway, we can get the conclusion that the maximum of PCC value will not appear at the position of IMF1 as long as there is vibration.

The EMD-PCC denoising method used in the φ-OTDR system can be obtained through two simulation experiments. The procedure is given as follows: (1) apply the EMD on the raw Rayleigh backscattering traces; (2) calculate the PCC between data and each IMFs (3) determine the maximum of the PCC; (4) if the PCC between data and the IMF1 is the maximum, only keep the residual component, otherwise, keep all the IMFs and residual components.

## 3. Experimental Setup and Discussion

**Figure 5:**
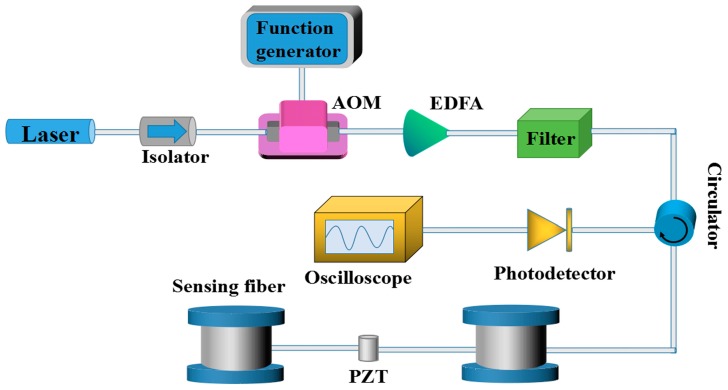
The experimental setup of the φ-OTDR system.

Figure 5 gives the experimental setup of the φ-OTDR system. The light source is an external cavity laser (ECL) operating at 1550.12 nm with narrow linewidth of about 3 KHz and maximum output power of about 10 mW. The isolator can effectively prevent the reflected light from returning to the laser to ensure the stability. The pulses are modulated by an acoustic optical modulator (AOM) which is driven by a function generator. An erbium-doped fiber amplifier (EDFA) is supposed to amplify the optical pulses and the ASE noise is removed by a tunable filter. The pulses are injected into sensing fiber with 2 km length via a circulator and a cylindrical PZT is located at the middle as a vibration source. The Rayleigh backscattering light is received by a photo detector (PD) and a high-speed oscilloscope is utilized to gather the electric signals.

**Figure 6:**
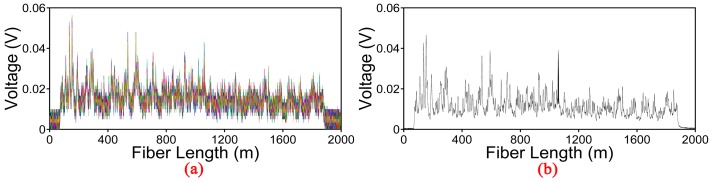
(**a**) Original Rayleigh backscattering curves for the vibration event of 100 Hz; (**b**) Denoised Rayleigh backscattering curves obtained by the EMD-PCC denoising method.

In our experiment, the width and repetition rate of the optical pulses are set to be 50 ns and 10 kHz respectively. The cylindrical PZT is driven by a sinusoidal signal produced by a function generator and the frequency of simulative vibration can be set from Hz to kHz. The number of Rayleigh backscattering curves is 1000 and it is collected by a high-speed oscilloscope with a sampling rate of 100 MHz. One thousand consecutive raw Rayleigh backscattering curves for the vibration source of 100 Hz are shown in Figure 6a. It is observed that the Rayleigh backscattering curves from the sensing fiber are jagged due to the coherent effect of multiple scattering centers within the probe pulse duration. Obviously, the vibration signal is submerged in strong background noise which cannot be identified by the raw curves. Then we apply the EMD-PCC denoising method and the result is shown in Figure 6b. There is obvious variation around the location of vibration at 1060 m. Meanwhile, all the DC components are retained at other location which is matched with the profile of the original Rayleigh backscattering curves. In order to observe the denoising effect intuitively, we remove the DC component and compare the result with some commonly-used denoising methods.

**Figure 7:**
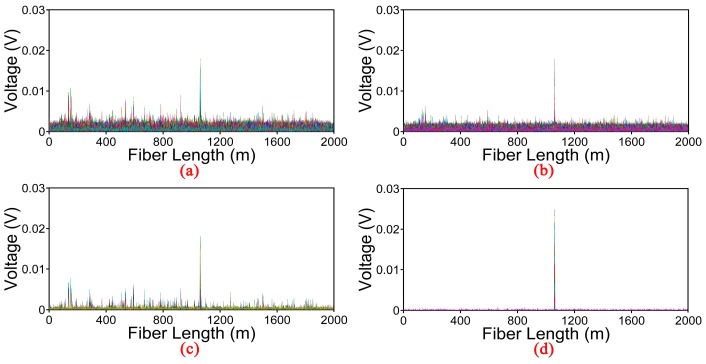
Location information processed by different methods. (**a**) Moving average and moving differential method; (**b**) EMD-soft denoising method; (**c**) Wavelet denoising method; (**d**) EMD-PCC denoising method.

Location information processed by several methods is shown in Figure 7. The moving average and moving differential method with average number of 20 is shown in Figure 7a. There is a peak at 1060 m which indicates the location of the vibration. However, the statement is unconvincing because the vibration signal is mixed with high background noise which may lead to misinformation. Figure 7b shows the location information processed by the EMD denoising method with soft thresholding (EMD-soft) [20]. The background noise is reduced but still remains at a high level. Figure 7c,d show the experiment results obtained by the wavelet denoising method [25] and the EMD-PCC denoising method respectively. We can find a clear peak corresponding to the vibration location both in the two figures. Furthermore, the voltage of vibration signal got by the EMD-PCC denoising method is higher and the background noise level is lower than the wavelet denoising method. In order to demonstrate the conclusion intuitively, the SNR of the location information is described as $\mathrm{SNR}=20\times \log_{10}\left(V_{signal}/{\mathrm{RMS}(V}_{\mathrm{noise}})\right)$, where V_signal_ and RMS (V_noise_) are the voltage of signal and the root-mean-square of the voltage of background noise respectively [26,27]. Here the V_signal_ is 0.018 V and the RMS(V_noise_) is 0.003 V through the moving average and moving differential method, so the SNR of the location information is 15.56 dB. The SNR of the location information raises to 18.30 dB obtained by the EMD-soft denoising method. When applying the wavelet denoising method, the SNR of the position information is equal to 24.54 dB. The SNR of the position information enormously increases to 42.52 dB when employing the EMD-PCC denoising method.

**Figure 8:**
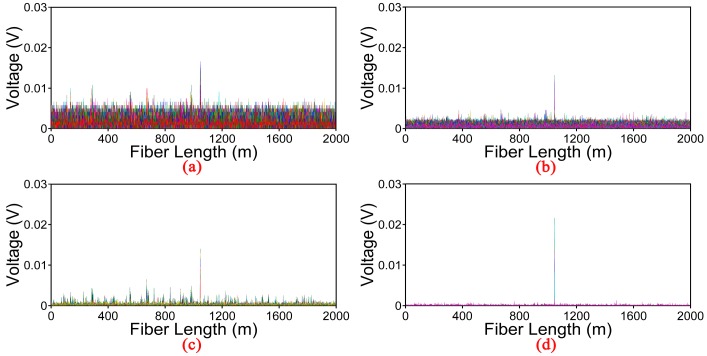
Location information processed by different methods for vibration event of 1.2 kHz. (**a**) Moving average and moving differential method; (**b**) EMD-soft denoising method; (**c**) Wavelet denoising method; (**d**) EMD-PCC denoising method.

Figure 8 shows the location information for the vibration source of 1.2 kHz obtained by different methods. The same conclusion is obtained when compared with the figure of 100 Hz. Although the wavelet denoising method is able to remove the noise, the SNR of location information should be further increased to meet the demands in the φ-OTDR system. 

**Figure 9:**
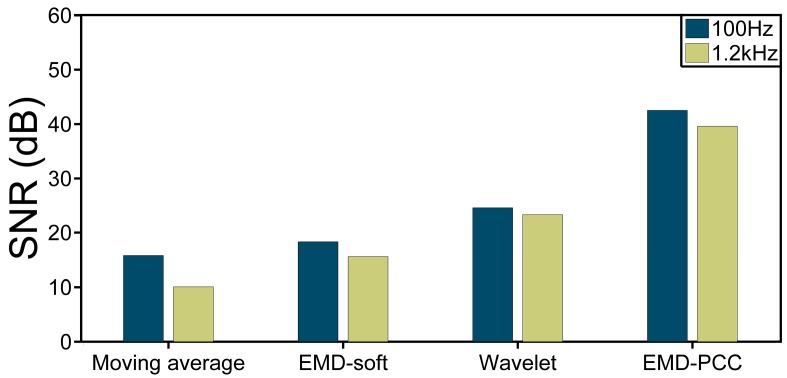
SNR of location information for 100 Hz and 1.2 kHz vibration events obtained by different methods.

When applying the EMD-PCC denoising method, the SNR of location information shows a prominent improvement. The SNR of position information for 100 Hz and 1.2 kHz vibration event discussed with the four methods is shown in Figure 9, which proves the viewpoint that the SNR of position information has a huge improvement by the EMD-PCC denoising method compared with the other three.

**Figure 10:**
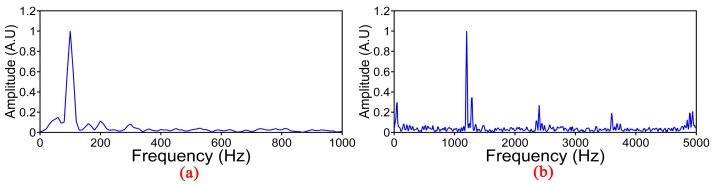
Frequency spectrum of two vibration events. (**a**) Frequency of 100 Hz; (**b**) Frequency of 1.2 KHz.

The frequency spectrum of 100 Hz and 1.2 kHz vibration events is shown in Figure 10a,b, respectively, by employing Fourier transform on the original Rayleigh backscattering traces. In Figure 10b, we can discover high-order harmonics at 2.4, 3.6 and 4.8 kHz with a fundamental frequency of 1.2 kHz, which may be caused by the large sinusoidal voltage applied on PZT [28].

## 4. Conclusions

In this paper, a new denoising method is proposed to reduce the time domain noise and increase the SNR for vibration measurements in φ-OTDR systems. The locations with or without the vibration event can be distinguished according to the PCC between noisy signal and each IMFs. Moreover, different procedures are performed on the positions with or without vibration in order to remove the background noise sufficiently and preserve the signal as much as possible. Experimental results show that the SNR of location information for the vibration source of 100 Hz and 1.2 kHz increases to 42.52 dB and 39.58 dB, respectively, which represents a great improvement when compared with the wavelet denoising method. This method is thus proved to an excellent solution for enhancing the performance of φ-OTDR systems. 

## References

[1] Dakin J.P., Pearce D.A.J., Strong A.P., Wade C.A. (1988). A novel distributed optical fibre sensing system enabling location of disturbances in a sagnac loop interferometer. Proc. SPIE.

[2] Sun Q.Z., Liu D.M., Wang J., Liu H.R. (2008). Distributed fiber-optic vibration sensor using a ring Mach-Zehnder interferometer. Opt. Commun..

[3] Yuan L.B., Ansari F. (1997). White-light interferometric fiber-optic distributed strain-sensing system. Sens. Actuators A.

[4] Hong X.B., Wu J., Zuo C., Liu F.S., Guo H.X., Xu K. (2011). Dual Michelson interferometers for distributed vibration detection. Appl. Opt..

[5] Zhang Z.Y., Bao X.Y. (2008). Distributed optical fiber vibration sensor based on spectrum analysis of Polarization-OTDR system. Opt. Express.

[6] Juarez J.C., Maier E.W., Choi K.N., Taylor H.F. (2005). Distributed fiber-optic intrusion sensor system. J. Lightwave Technol..

[7] Martins H.F., Martin-Lopez S., Corredera P., Filograno M.L., Frazao O., Gonzalez-Herraez G. (2013). High visibility phase-sensitive optical time domain reflectometer for distributed sensing of ultrasonic waves. Proc. SPIE.

[8] Masoudi A., Belal M., Newson T.P. (2013). A distributed optical fibre dynamic strain sensor based on phase-OTDR. Meas. Sci. Technol..

[9] Dong Y.K., Ba D.X., Jiang T.F., Zhou D.W., Zhang H.Y., Zhu C.Y., Lu Z.W., Li H., Chen L., Bao X.Y. (2013). High-spatial-resolution fast BOTDA for dynamic strain measurement based on differential double-pulse and second-order sideband of modulation. IEEE Photonics J..

[10] Peng F., Wu H., Jia X.H., Rao Y.J., Wang Z.N., Peng Z.P. (2014). Ultra-long high-sensitivity Φ-OTDR for high spatial resolution intrusion detection of pipelines. Opt. Express.

[11] Zhou L., Wang F., Wang X.C., Pan Y., Sun Z.Q., Hua J., Zhang X.P. (2015). Distributed Strain and Vibration Sensing System Based on Phase-Sensitive OTDR. IEEE Photonics Technol. Lett..

[12] Tu G.J., Zhang X.P., Zhang Y.X., Fan Z., Xia L., Nakarmi B. (2015). The Development of an Φ-OTDR System for Quantitative Vibration Measurement. IEEE Photonics Technol. Lett..

[13] Healey P. (1984). Fading in heterodyne OTDR. Electron. Lett..

[14] Lu Y.L., Zhu T., Chen L., Bao X.Y. (2010). Distributed Vibration Sensor Based on Coherent Detection of Phase-OTDR. J. Lightwave Technol..

[15] Qin Z.G., Chen L., Bao X.Y. (2012). Wavelet Denoising Method for Improving Detection Perforamance of Distributed Vibration Sensor. IEEE Photonics Technol. Lett..

[16] Zhu T., Xiao X.H., He Q., Diao D.M. (2013). Enhancement of SNR and Spatial Resolution in φ-OTDR System by Using Two-Dimensional Edge Detection Method. J. Lightwave Technol..

[17] Li Q., Zhang C.X., Li L.J., Zhong X. (2014). Signal-to-noise ratio enhancement of Phase -sensitive optical time-domain reflectometry based on power spectrum analysis. Opt. Eng..

[18] Huang N.E., Shen Z., Long S.R., Wu M.C., Shih H.H., Zheng Q., Yen N.C., Tung C.C., Liu H.H. (1998). The empirical mode decomposition and the Hilbert spectrum for nonlinear and non-stationary time series analysis. Proc. R. Soc. Lond. A.

[19] Rilling G., Flandrin P., Goncalvès P. On Empirical Mode Decomposition and Its Algorithms. Proceedings of the IEEE-EURASIP Workshop on Nonlinear Signal and Image Processing (NSIP 2003).

[20] Boudraa A.O., Cexus L.C., Saidi Z. (2004). EMD-Based Signal Noise Reduction. Int. J. Signal Process.

[21] Omitaomu O.A., Protopopescu V.A., Ganguly A.R. (2011). Empirical Mode Decomposition Technique with Conditional Mutual Information for Denoising Operational Sensor Data. IEEE Sens. J..

[22] Hassan M., Boudaoud S., Terrien J., Karlsson B., Marque C. (2011). Combination of Canonical Correlation Analysis and Empirical Mode Decomposition Applied to Denoising the Labor Electrohysterogram. IEEE Trans. Biomed. Eng..

[23] Pal S., Mitra M. (2012). Empirical mode decomposition based ECG enhancement and QRS detection. Comput. Boil. Med..

[24] Mukaka M.M. (2012). Statistics Corner: A guide to appropriate use of Correlation coefficient in medical research. Malawi Med. J..

[25] Peng Z.K., Chu F.L. (2004). Application of the wavelet transform in machine condition monitoring and fault diagnostics: A review with bibliography. Mech. Syst. Signal Process.

[26] Fang G., Xu T., Feng S., Li F. (2015). Phase-sensitive Optical Time Domain Reflectometer Based on Phase Generated Carrier Algorithm. J. Lightwave Technol..

[27] Wang Z., Zhang L., Wang S., Xue N., Peng F., Fan M., Sun W., Qian X., Rao J., Rao Y. (2016). Coherent Φ-OTDR based on I/Q demodulation and homodyne detection. Opt. Express.

[28] Ren M.Q., Lu P., Chen L., Bao X.Y. (2016). Study of Φ-OTDR Stability for Dynamic Strain Measurement in Piezoelectric Vibration. Photonic Sens..

